# RNF20 is required for male fertility through regulation of H2B ubiquitination in the Sertoli cells

**DOI:** 10.1186/s13578-023-01018-2

**Published:** 2023-04-06

**Authors:** Fengling Lai, Haoyu Wang, Xinyue Zhao, Kangning Yang, Le Cai, Mengxin Hu, Lan Lin, Xizhong Xia, Wei Li, Hanhua Cheng, Rongjia Zhou

**Affiliations:** 1 Hubei Key Laboratory of Cell Homeostasis, College of Life Sciences, Renmin Hospital of Wuhan University, Wuhan University, Wuhan, 430072 China; 2 Guangzhou Women and Children’s Medical Center, Guangzhou, 510623 China

**Keywords:** Male infertility, Sertoli cells, Cell adhesion, Spermatogenesis, RNF20

## Abstract

**Background:**

Spermatogenesis depends on the supporting of the Sertoli cells and their communications with germ cells. However, the regulation of crosstalk between the Sertoli cells and germ cells remains unclear.

**Results:**

In this report, we used conditional knockout technology to generate the Sertoli cells-specific knockout of *Rnf20* in mice. The *Amh-Rnf20*^*−/−*^ male mice were infertile owing to spermatogenic failure that mimic the Sertoli cell-only syndrome (SCOS) in humans. Knockout of *Rnf20* resulted in the H2BK120ub loss in the Sertoli cells and impaired the transcription elongation of the *Cldn11*, a gene encoding a component of tight junction. Notably, RNF20 deficiency disrupted the cell adhesion, caused disorganization of the seminiferous tubules, and led to the apoptotic cell death of both spermatogonia and spermatocytes in the seminiferous tubules.

**Conclusions:**

This study describes a *Rnf20* knockout mouse model that recapitulates the Sertoli cell-only syndrome in humans and demonstrates that RNF20 is required for male fertility through regulation of H2B ubiquitination in the Sertoli cells.

**Supplementary Information:**

The online version contains supplementary material available at 10.1186/s13578-023-01018-2.

## Introduction

In humans, infertility is common and affects approximately 17% of reproductive-aged couples worldwide [1–4]. Near 50% of infertility cases are caused by male factors [5], which include a reduced number of spermatozoa, low sperm quality, and spermatogenesis failure. Azoospermia is an important factor affecting male infertility [6]. The Sertoli cell-only syndrome (SCOS) is one of the severe disorders which causes male infertility and is usually azoospermic. The typical features of the SCOS are germ cell aplasia, a reduced testicular volume, and only Sertoli cells line in the seminiferous tubules [7]. Several causal factors have been proposed for the pathogenic mechanisms underlying the SCOS, including chromosomal abnormality [8, 9], sex chromosome disorders, for example, Klinefelter syndrome [10], Y chromosome microdeletions in AZFs [11–14], mutations of candidate genes [15–19], and dysregulation of gene expression [20, 21]. Recently, *Mast4* [22] and *Pramef12* [23] have been identified as essential genes for spermatogenesis in mice, and knockout of the genes recapitulates the SCOS in humans. In addition, copy number variations and endocrine processes could also be causal for the SCOS [10, 24]. Thus, in humans, the SCOS is a clinically and genetically heterogeneous group of disorders for male infertility and its pathogenic mechanisms remain elusive.

Spermatogenesis is a precisely regulated and complex process of development and differentiation, which is typically divided into three phases: proliferation of spermatogonia stem cells through mitosis, differentiation of spermatocytes to spermatids through meiotic cell division, and spermiogenesis [25]. During spermatogenesis in testis, the supporting cells, the Sertoli cells, in the seminiferous tubules, provide an essential niche in structural, immunological, and nutritional support for germ cell development [26]. The Sertoli cells are located on the basement membrane of the seminiferous tubules and function not only as nurse cells in the testis, but also establish an immune-privileged environment of the blood-testis-barrier (BTB) for spermatogenesis [26–28]. Thus, the Sertoli–germ cell crosstalk is crucial for spermatogenesis; however, the regulation mechanisms of their interactions remain elusive. As a major component of the BTB, CLDN11 contributes to the tight junction between the Sertoli cells and spermatogonial stem cells (SSCs), which plays an essential role during spermatogenesis [29–31]. Deficiency of *Cldn11* in mice resulted in loss of tight junction integrity and spermatogenesis did not proceed beyond the spermatocyte stage [32]. In addition, the impairment of spermatogenesis and male infertility were involved in an abnormal distribution of *Cldn11* expression in the Sertoli cells [33, 34]. However, the epigenetic regulation of CLDN11 expression in the Sertoli cells remains unknown.

The E3 ubiquitin ligase RNF20 (ring finger protein 20) is critical for mono-ubiquitination of histone H2B at lysine 120 (H2BK120ub) in mammals [35, 36]. It is now clear that RNF20 and H2BK120ub play vital roles in some of the most fundamental biological processes and global knockout of *Rnf20* in mice resulted in very early embryonic lethality [37]. RNF20 was implicated in the regulation of double-strand break repair [38], suppression of tumorigenesis [39], cell differentiation [40, 41], homologous recombination [42], mRNA splicing [43], adipose tissue development [44], and meiotic recombination and spermatogenesis [45]. Notably, RNF20 was a reliable indicator of transcription activity and contributed to transcriptional elongation [43, 46, 47]. Although considerable efforts have been made for RNF20, the biological function and underlying mechanisms of RNF20 in the Sertoli cells in regulation of spermatogenesis remain unclear.

In the present study, we created a *Rnf20* conditional knockout in the Sertoli cells and find that deficiency of *Rnf20* leads to male infertility owing to spermatogenic failure in mice, recapitulating pathological features of the SCOS in humans. *Rnf20* knockout results in the H2BK120ub loss in the Sertoli cells and thus impairs the transcription elongation of the *Cldn11* gene in the Sertoli cells, which is essential for the BTB establishment through the cell junction. RNF20 deficiency in the Sertoli cells disrupts the cell adhesion, causes disorganization of the seminiferous tubules, and leads to the loss of both the spermatogonia and spermatocytes in the seminiferous tubules by apoptosis pathway. These molecular and cellular events provide new insights to the male infertility.

## Results

### The Sertoli cells-specific knockout of ***Rnf20*** results in male infertility

**Fig. 1:**
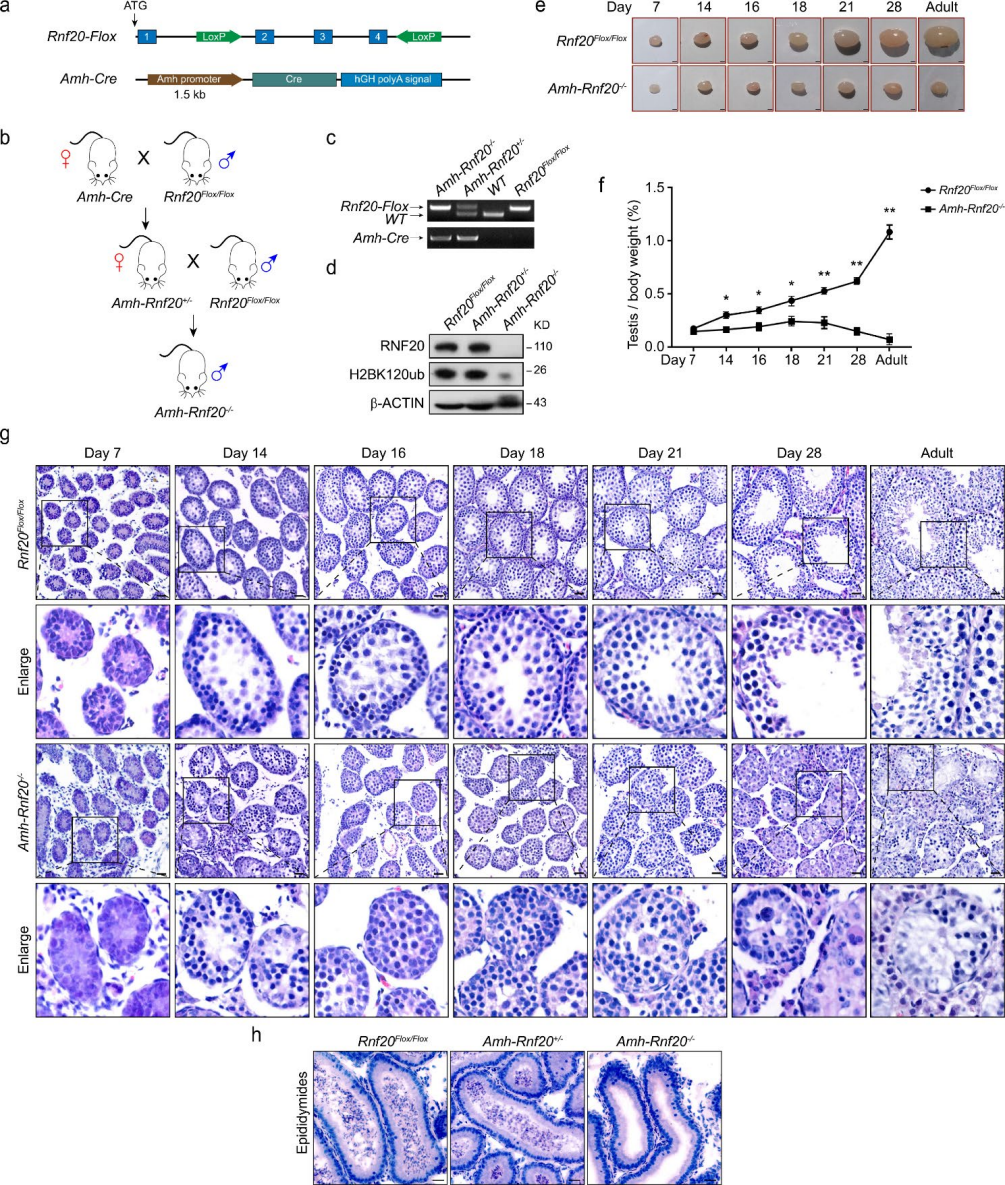
Spermatogenic failure in the *Amh-Rnf20*^*−/−*^ mice. **a** Schematic diagram for the construction for making the *Rnf20*^*Flox/Flox*^ and the *Amh-Cre* mice. **b** Flow chart of breeding to generate the *Amh-Rnf20*^*−/−*^ mice. **c** Genotyping of the *Rnf20* conditional knockout mice. *Rnf20-Flox*, *Rnf20*^*Flox/Flox*^; WT, wild type; *Amh-Cre*, *Amh* promoter-induced conditional knockout. **d** Western blot analysis of the protein levels of RNF20 and the ubiquitylation of histone H2BK120 in adult mice of 3 types of genotypes. β-ACTIN was used as an internal control. **e** Comparison the size of testes between the *Rnf20*^*Flox/Flox*^ and the *Amh-Rnf20*^*−/−*^ in adult and at 7, 14, 16, 18, 21, and 28 days after birth. Scale bar, 100 μm. **f** Relative weight of testis to body weight in mice between genotypes of the *Rnf20*^*Flox/Flox*^ and the *Amh-Rnf20*^*−/−*^ (n = 3), *, p < 0.05, **, p < 0.01. **g** H&E staining of testes in the *Rnf20*^*Flox/Flox*^ and the *Amh-Rnf20*^*−/−*^ in adult and at 7, 14, 16, 18, 21, and 28 days after birth. The black square in the panels corresponds to the enlarged panels. Scale bar, 100 μm. **h** H&E staining of epididymides in adult mice. Scale bar, 100 μm

To investigate the function of *Rnf20* in the Sertoli cells during spermatogenesis, the *Rnf20*^*Flox/Flox*^ mice were used [48]. The Lox P sites were inserted between exon 2–4 of *Rnf20*, where *Rnf20* was knocked out through disrupted the ORF of mRNA (Fig. 1a). The *Amh-Rnf20*^*−/−*^ mice were produced by crossing the *Rnf20*^*Flox/Flox*^ with the *Amh-Cre* mice (Fig. 1b, c). Western blot analysis confirmed that the protein levels of RNF20 and H2BK120ub were significantly decreased in the testes of the *Amh-Rnf20*^*−/−*^ mice compared with those in the *Amh-Rnf20*^*+/−*^ and the *Rnf20*^*Flox/Flox*^ mice (Fig. 1d). The morphology observations showed that the testis size in the *Amh-Rnf20*^*−/−*^ mice from the day 14 after birth to adult was significantly smaller than those in the *Rnf20*^*Flox/Flox*^ mice (Fig. 1e, f), indicating testis development was impaired in the *Amh-Rnf20*^*−/−*^ mice. Further histology analysis of testes at different stages of spermatogenesis showed that there were no round or elongated spermatids in the seminiferous tubules of the *Amh-Rnf20*^*−/−*^ mice and no spermatozoa were detected in the seminiferous tubules, even there were only Sertoli cells and a few of germ cells existed in the seminiferous tubules, while sperm cells were produced gradually in the *Rnf20*^*Flox/Flox*^ mice (Fig. 1g). In addition, histological analysis of the epididymal lumens of the *Amh-Rnf20*^*−/−*^, the *Amh-Rnf20*^*+/−*^, and the *Rnf20*^*Flox/Flox*^ mice showed that mature spermatozoa were absent in the epididymal lumens in the *Amh-Rnf20*^*−/−*^ male mice (Fig. 1h). These observations were consistent with the fact that the *Amh-Rnf20*^*−/−*^ male mice were infertile when mating with wild-type female mice. Nevertheless, the knockout of the *Amh-Rnf20*^*−/−*^ had no detectable effect on ovary development and oogenesis (Additional file 1: Fig. S1). Together, these data indicated that the Sertoli cells-specific knockout of *Rnf20* in mice caused male infertility owing to spermatogenic failure, recapitulating pathological features of the SCOS in humans.

### RNF20 deficiency results in H2BK120ub loss in the Sertoli cells

**Fig. 2:**
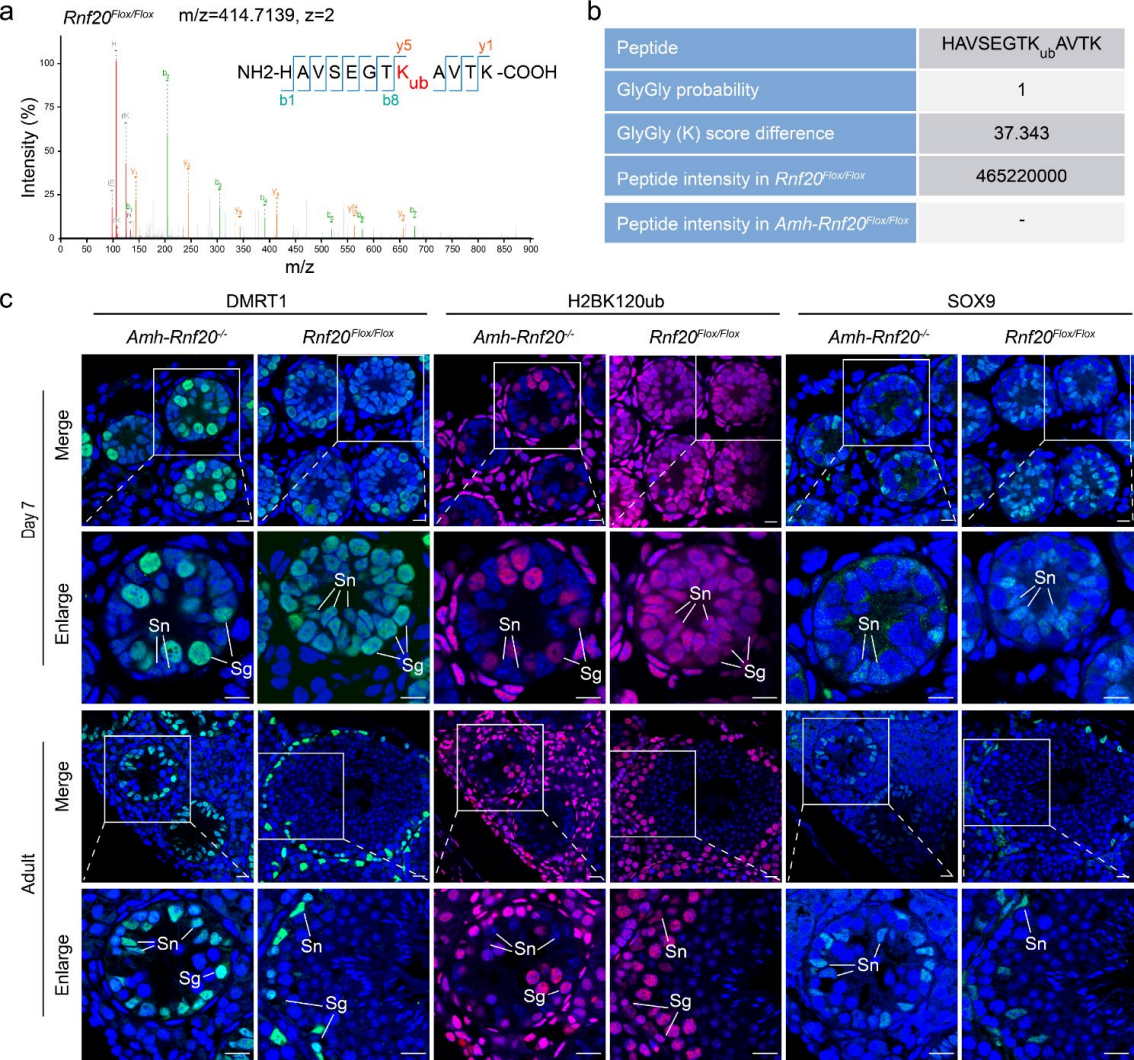
H2BK120ub is deficient in the Sertoli cells in the *Amh-Rnf20*^*−/−*^ mice. **a** Mass spectrometry detection of the ubiquitination of the H2B at K120 with the peptide HAVSEGTK(120)AVTK in the *Rnf20*^*Flox/Flox*^. The MQ software was used to analyze the data from mass spectrometry. X axis, m/z; Y axis, the intensity of ions; y, the C-terminal fragment ion (Y series). **b** Ubiquitinated peptide information at the position of the K120 in the *Rnf20*^*Flox/Flox*^. The ubiquitination modification site was not detected in the *Amh-Rnf20*^*−/−*^. **c** Immunofluorescent analysis of SOX9, H2BK120ub, and DMRT1 on serial paraffin-sections in the *Rnf20*^*Flox/Flox*^ and the *Amh-Rnf20*^*−/−*^ testes at 7 days after birth and adult mice. The nuclei were stained with DAPI. TRITC signals represent the localization of H2BK120ub, while FITC signals showed the localization of SOX9 or DMRT1. The white squares in the panels correspond to the enlarged panels. Sn, Sertoli cells; Sg, spermatogonia. Scale bar, 10 μm

RNF20 is an E3 ubiquitin ligase critical for mono-ubiquitination of histone H2B at lysine 120 [35, 36]. To explore ubiquitination change of histone H2B in the Sertoli cells of *Rnf20* knockout, mass spectrometry was used to detect the levels of H2B ubiquitination in the *Amh-Rnf20*^*−/−*^ and the *Rnf20*^*Flox/Flox*^ mice. H2BK120ub was remarkably detected in the Sertoli cells in the *Rnf20*^*Flox/Flox*^, whereas no signal at the site on H2B in the Sertoli cells in the *Amh-Rnf20*^*−/−*^ was observed (Fig. 2a, b). Further immunofluorescence assays were conducted to verify the levels of H2BK120ub in different types of cells in the *Rnf20*^*Flox/Flox*^ and the *Amh-Rnf20*^*−/−*^ mouse testes. SOX9 is a marker of the Sertoli cells, while DMRT1 is a marker for both the Sertoli cells and spermatogonia [49, 50], which were used to identify the expression localization of H2BK120ub in the Sertoli cells. Immunofluorescence analysis using these markers showed that knockout of *Rnf20* led to the loss of H2BK120ub in the Sertoli cells during spermatogenesis (Fig. 2c; Additional file 1: Fig. S2). These results suggested that *Rnf20* knockout resulted in the H2BK120ub loss in the Sertoli cells.

### RNF20 deficiency in the Sertoli cell impairs the ***Cldn11*** transcription

**Fig. 3:**
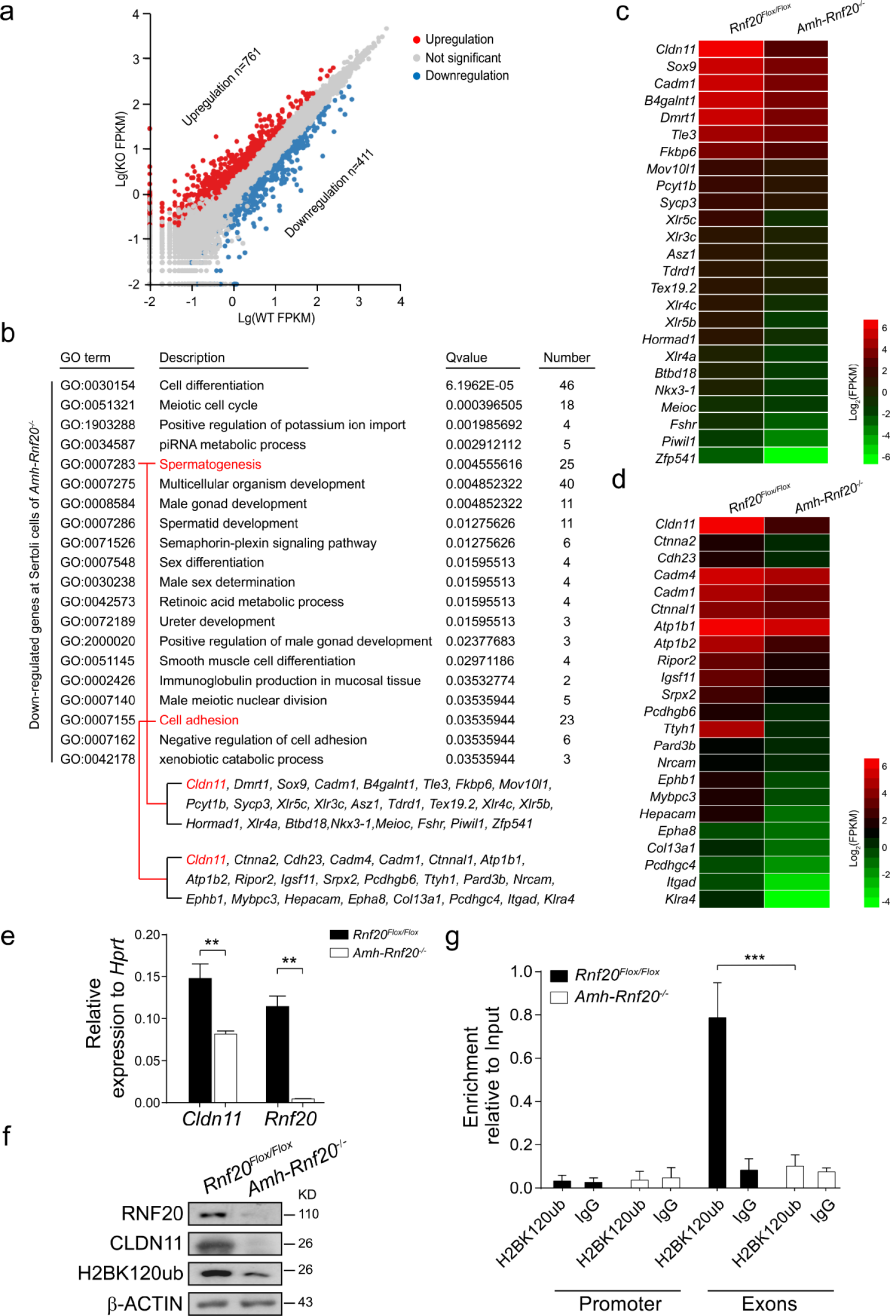
RNF20 deficiency in Sertoli cells impairs the *Cldn11* transcription. **a** Scatterplots of differentially expressed genes. Red scatter, genes with significant up-regulated; blue scatter, genes with significant down-regulated; gray scatter, genes with no significant difference. X axis, Lg (WT FPKM) in the *Rnf20*^*Flox/Flox*^; Y axis, Lg (KO FPKM) in the *Amh-Rnf20*^*−/−*^. **b** Gene ontology (GO) terms analysis of down-regulated genes in the Sertoli cells of the *Amh-Rnf20*^*−/−*^ testes. **c, d** Heatmaps showing the expression levels of down-regulated genes in the terms spermatogenesis (**c**) and cell adhesion (**d**) in the Sertoli cells of the *Rnf20*^*Flox/Flox*^ and the *Amh-Rnf20*^*−/−*^. Color bar, Log_2_ (FPKM). **e** Quantitative real-time PCR analysis of the genes *Rnf20* and *Cldn11*. The expression levels of the genes were related to *Hprt* expression. Relative levels, 2^−ΔCt^; T-tests were performed. *, p < 0.05, **, p < 0.01. **f** Western blot analysis of the expression levels of RNF20, CLDN11, and H2BK120ub proteins in adult mice. β-ACTIN was used as an internal control. **g** ChIP-PCR assays. The antibody specific for H2BK120ub was used in the ChIP analysis and primers were designed in the regions of promoter and exons of *Cldn11* in the testes of the *Rnf20*^*Flox/Flox*^ and the *Amh-Rnf20*^*−/−*^. The black graphs indicated the enriched levels in the *Rnf20*^*Flox/Flox*^ mice, while the white graphs indicated the levels in the *Amh-Rnf20*^*−/−*^ mice

To examine potential roles of *Rnf20* in regulation of gene transcription involved in spermatogenesis, we performed RNA-Seq analysis of the Sertoli cells isolated from the *Rnf20*^*Flox/Flox*^ and *Amh-Rnf20*^*−/−*^ mice at 7 days after birth. Over 1172 differentially expressed genes (DEGs) were detected, based on both |Log_2_FC|>1 (FC, fold change) and FDR < 0.001 (FDR, false discovery rate), in the Sertoli cells of the *Amh-Rnf20*^*−/−*^ mice compared with those of the *Rnf20*^*Flox/Flox*^ mice. Of which 761 genes were upregulated and 411 genes were down-regulated (Fig. 3a). Gene Ontology (GO) analysis in biological process of the down-regulated genes showed an enrichment in cell differentiation, meiotic cell cycle, spermatogenesis, and cell adhesion (Fig. 3b). Of them, *Cldn11*, a gene encoded the major component of the BTB and tight junction, was associated with both GO processes, spermatogenesis and cell adhesion, and was significantly down-regulated in the Sertoli cells of the *Amh-Rnf20*^*−/−*^ mice compared with those of the *Rnf20*^*Flox/Flox*^ mice (Fig. 3b-d). Quantitative real-time PCR confirmed the down-regulation of *Cldn11* at mRNA levels in testes of adult mice (Fig. 3e). CLDN11 protein was also down-regulated in the testes of adult mice of the *Amh-Rnf20*^*−/−*^ in comparison with those of the *Rnf20*^*Flox/Flox*^ mice (Fig. 3f). Further ChIP-PCR analysis showed that H2BK120ub was enriched in the exons regions, but seldomly in the promoter regions of the *Cldn11* gene in the *Rnf20*^*Flox/Flox*^ testes (Fig. 3g; Additional file 1: Fig. S3), whereas it was hardly ever detected in both the exons and promoter regions of the *Cldn11* owing to the lack of H2BK120ub in the *Amh-Rnf20*^*−/−*^ testes (Fig. 3f, g; Additional file 1: Fig. S3). These data indicated that *Rnf20* knockout in the Sertoli cells impaired the transcription elongation of *Cldn11* in the Sertoli cells owing to the H2BK120ub loss.

### RNF20 deficiency disrupts the cell adhesion and causes disorganization of the seminiferous tubules

**Fig. 4:**
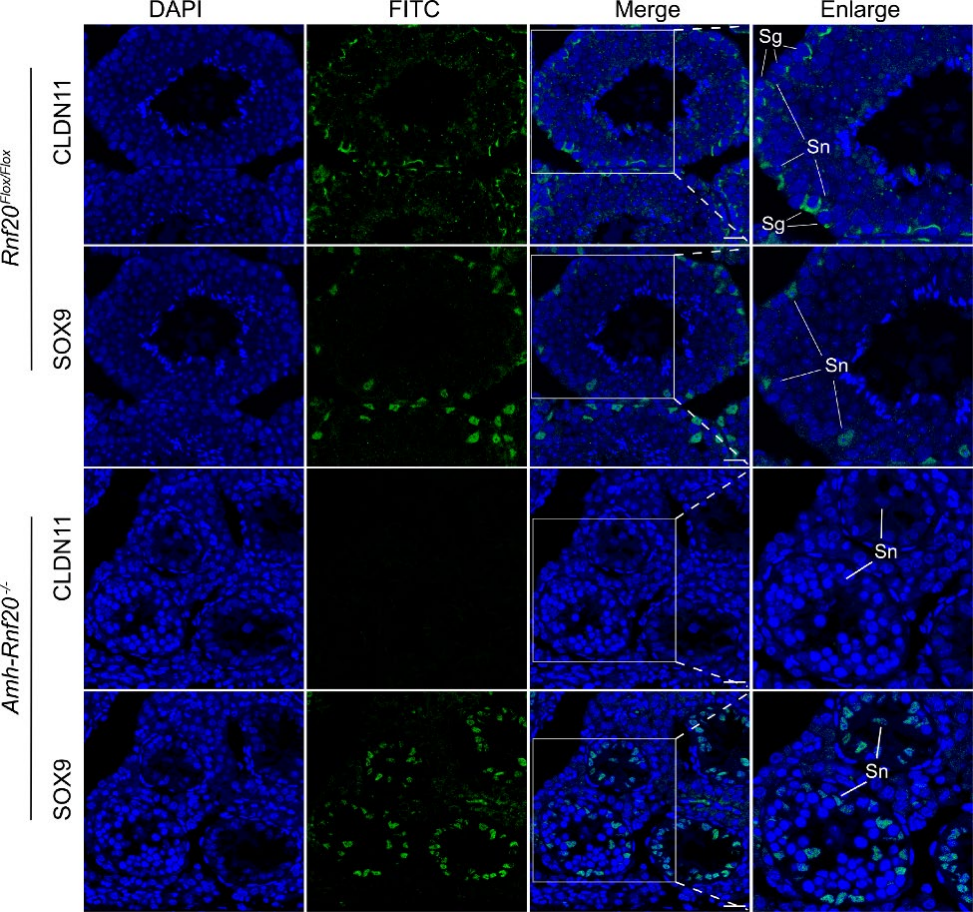
Knockout of *Rnf20* in the Sertoli cells impairs the CLDN11 expression and disrupts the distribution in the Sertoli cells in the seminiferous tubules in adult mice. Immunofluorescence analysis of the CLDN11 protein on testis sections in the *Rnf20*^*Flox/Flox*^ and the *Amh-Rnf20*^*−/−*^mice. The nuclei were stained with DAPI. FITC signals represent the localization of CLDN11. SOX9, a marker for the Sertoli cells, indicates the distribution of the Sertoli cells in the seminiferous tubules. The white squares in the panels correspond to the enlarged panels. Sn, Sertoli cells; Sg, spermatogonia; SOX9, a marker for the Sertoli cells. Scale bar, 25 μm

**Fig. 5:**
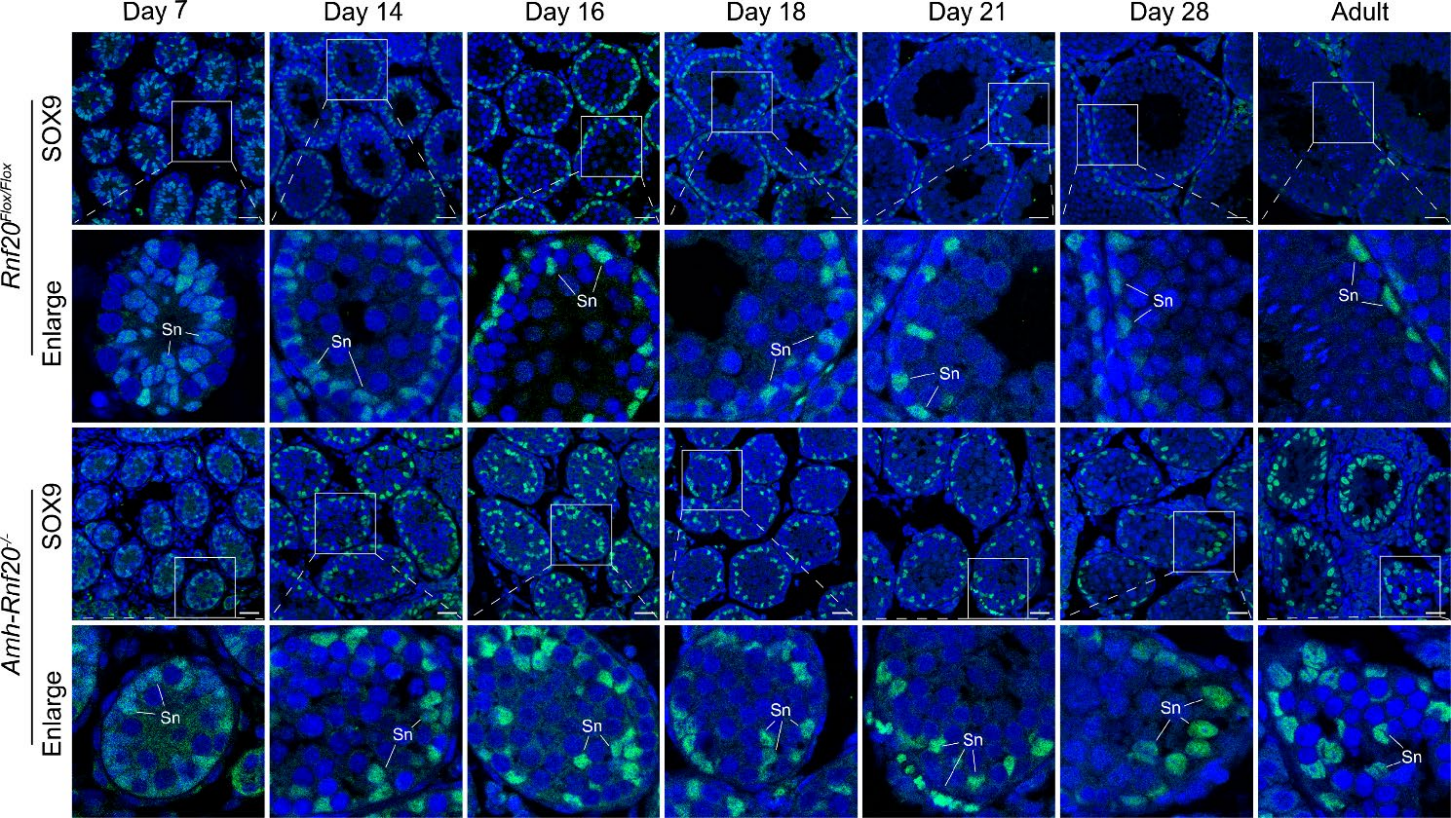
Knockout of *Rnf20* disrupts the distribution of the Sertoli cells in the seminiferous tubules from 14 days to adult. Immunofluorescence analysis showed the localization of the Sertoli marker SOX9 in the seminiferous tubules in the testes of the *Rnf20*^*Flox/Flox*^ and the *Amh-Rnf20*^*−/−*^ in adults and at 7, 14, 16, 18, 21, and 28 days after birth. The nuclei were stained with DAPI. The green fluorescence signals represent the localization of SOX9. The white squares in the panels correspond to the enlarged panels. Sn, Sertoli cells. Scale bar, 25 μm

**Fig. 6:**
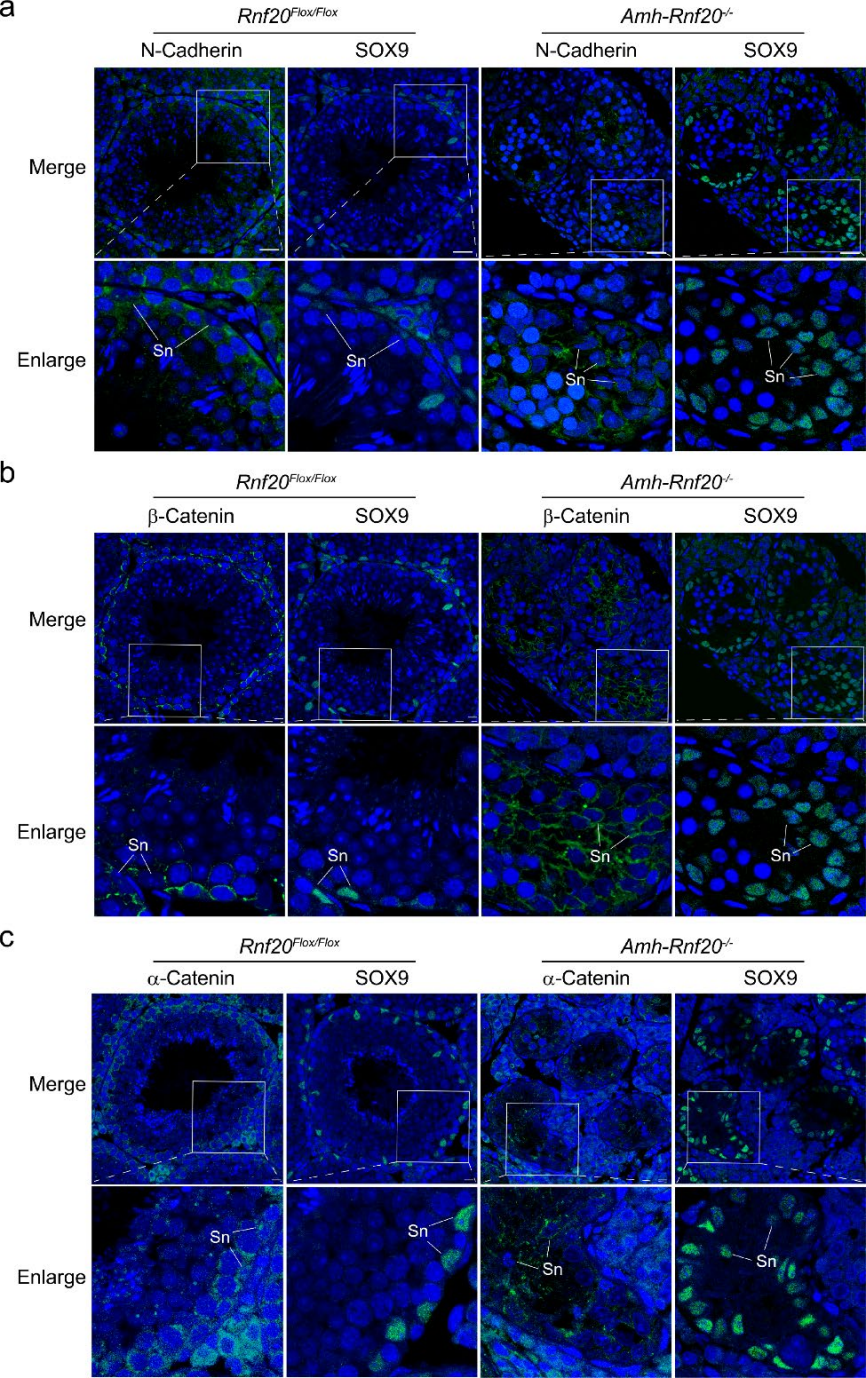
Knockout of *Rnf20* in the Sertoli cells disrupts the cell adhesion in the seminiferous tubules in adult mice. **a** Immunofluorescent analysis of N-Cadherin and SOX9 on the serial paraffin-sections in the *Rnf20*^*Flox/Flox*^ and the *Amh-Rnf20*^*−/−*^ testes. **b** Immunofluorescent analysis of β-Catenin and SOX9 on the serial paraffin-sections in the *Rnf20*^*Flox/Flox*^ and the *Amh-Rnf20*^*−/−*^ testes. **c** Immunofluorescent analysis of α-Catenin and SOX9 on the serial paraffin-sections in the *Rnf20*^*Flox/Flox*^ and the *Amh-Rnf20*^*−/−*^ testes. The nuclei were stained with DAPI. The white squares in the panels correspond to the enlarged panels. Sn, Sertoli cells. Scale bar, 25 μm

As the deficiency of H2BK120ub in the *Cldn11* gene region led to a decrease level of CLDN11, we further investigated CLDN11 expression in the seminiferous tubules and possible dysfunction of the cell adhesion. Immunofluorescence analysis showed that CLDN11 was hardly ever observed in testes of adult mice of the *Amh-Rnf20*^*−/−*^. In contrast, it was expressed in the peripheral regions surrounding the Sertoli cells and spermatogonia in the seminiferous tubules in the *Rnf20*^*Flox/Flox*^ testes (Fig. 4), suggesting a role of CLDN11 via H2BK120ub in the cell adhesion in the seminiferous tubules. Further, the Sertoli cells, as indicated by the marker SOX9, were arranged in the peripheral regions of the seminiferous tubules in the *Rnf20*^*Flox/Flox*^ testes, whereas in the *Amh-Rnf20*^*−/−*^ testes, the Sertoli cells were spread inward from the peripheral seminiferous tubules and some were observed in the center of the seminiferous tubules (Fig. 4). Further immunofluorescence analysis of testes at different days after birth (7, 14, 16, 18, 21, 28 days) and in adults showed that the abnormal distribution of the Sertoli cells in the seminiferous tubules was obvious from 14 days to adult (Fig. 5). The disorganized distribution of the Sertoli cells owing to down-regulation of the CLDN11 expression suggested a disruption of the cell adhesion. We further analyzed expression pattern of key cell-adhesion molecules, α-Catenin, β-Catenin, and N-Cadherin, in testes of adult mice. Immunofluorescence analysis of testis showed that these cell-adhesion molecules had a similar localization to that of CLDN11 in the peripheral regions surrounding the Sertoli cells in the seminiferous tubules in the *Rnf20*^*Flox/Flox*^ testes, as indicated by the marker SOX9 (Fig. 6). In contrast, in the *Amh-Rnf20*^*−/−*^ testes, these cell-adhesion molecules were spread inward from the peripheral seminiferous tubules and some were also detected in the center of the seminiferous tubules (Fig. 6). These data suggested that knockout of *Rnf20* disrupted the cell adhesion and led to disorganization of the seminiferous tubules.

### Apoptosis increases in the germ cells of the ***Amh-Rnf20***^***−/−***^ mice

**Fig. 7:**
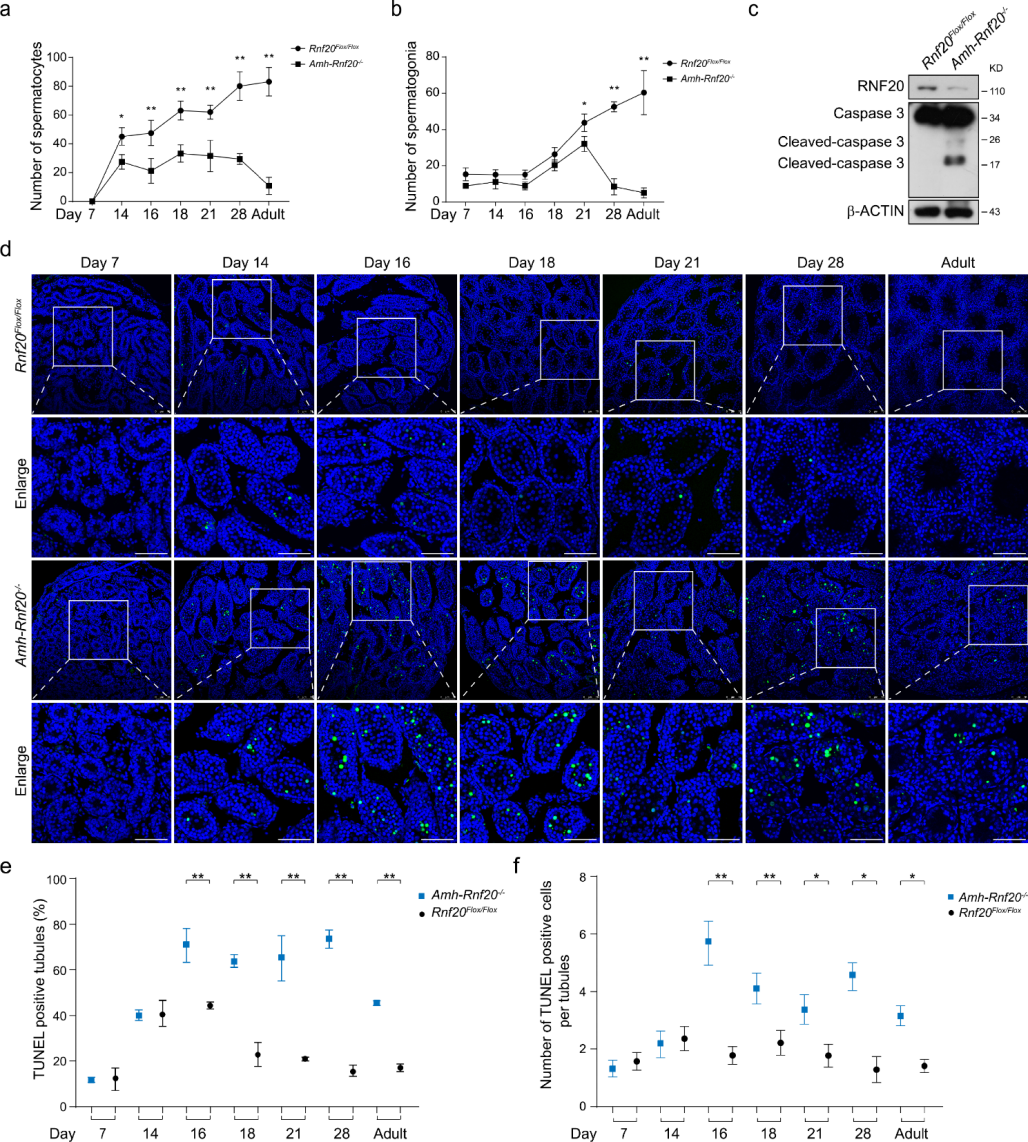
Apoptosis levels in the germ cells of the *Amh-Rnf20*^*−/−*^ testes were increased significantly during spermatogenesis. **a, b** Comparison of the number of spermatocytes (**a**) and spermatogonia (**b**) in the seminiferous tubules between the *Rnf20*^*Flox/Flox*^ and the *Amh-Rnf20*^*−/−*^ in adults and at 7, 14, 16, 18, 21, and 28 days after birth. Y axis, the number of germ cells; X axis, postnatal days. Black dots on the lines represent the *Rnf20*^*Flox/Flox*^, black squares on the lines represent the *Amh-Rnf20*^*−/−*^. *, p < 0.05, **, p < 0.01. **c** Western blot analysis of the expression levels of RNF20 and Caspase3 in testes of adult mice of the *Rnf20*^*Flox/Flox*^ and the *Amh-Rnf20*^*−/−*^. β-ACTIN was used as an internal control. **d** TUNEL assays were used to analyze the apoptotic signals in testes of the *Rnf20*^*Flox/Flox*^ and the *Amh-Rnf20*^*−/−*^ in adults and at 7, 14, 16, 18, 21, and 28 days after birth. The nuclei were stained with DAPI. The green fluorescence showed the apoptosis signals. The white squares in the panels correspond to the enlarged panels. Scale bar, 75 μm. **e** Statistical analysis of the proportion of TUNEL-positive seminiferous tubules. *, p < 0.05, **, p < 0.01. **f** Statistical analysis of the cell number with TUNEL-positive signals in the seminiferous tubules. Black dots represent the *Rnf20*^*Flox/Flox*^, blue squares indicate the *Amh-Rnf20*^*−/−*^. *, p < 0.05, **, p < 0.01

To further explore possible loss of the germ cells in the seminiferous tubules during spermatogenesis owing to the RNF20 deficiency in the Sertoli cells, we tested the levels of apoptosis in testes at different developmental stages. Statistical analysis showed that the number of the spermatocytes in the seminiferous tubules of the *Amh-Rnf20*^*−/−*^ mice was significantly decreased, compared with that in the *Rnf20*^*Flox/Flox*^ mice from day 14 after birth to adult (Fig. 7a). Moreover, the number of the spermatogonia in the seminiferous tubules was significantly reduced in the *Amh-Rnf20*^*−/−*^ mice from day 21 after birth to adult (Fig. 7b). In addition, Western blot analysis showed that the levels of cleaved-caspase 3 were up-regulated in the testes of adult mice of the *Amh-Rnf20*^*−/−*^ compared to those of the *Rnf20*^*Flox/Flox*^ mice (Fig. 7c). Further, we tested the TUNEL-positive signals during the progress of spermatogenesis. The TUNEL analysis confirmed that the apoptosis in testes was up-regulated in the *Amh-Rnf20*^*−/−*^ mice (Fig. 7d). Statistical analysis indicated that both numbers of TUNEL-positive cells in per tubule and the TUNEL-positive tubules were significantly increased from day 16 after birth to adult (Fig. 7e, f). Taken together, these results suggested that RNF20 deficiency caused the loss of germ cells in the seminiferous tubules by the apoptosis pathway.

## Discussion

In humans, male infertility is common and affects approximately 7% of the male population worldwide [51]. Because of the complex pathogenetic mechanisms, the aetiology of male infertility remains elusive in humans. Here, we used conditional knockout technology to investigate spermatogenic failure and underlying molecular mechanisms. Our study provides a mouse model of the *Rnf20* knockout, that recapitulates the Sertoli cell-only syndrome in humans, a serious condition for male infertility. The Sertoli cells-specific knockout of *Rnf20* causes male infertility. RNF20 deficiency leads to the H2BK120ub loss which impairs the transcription elongation of the *Cldn11* gene in the Sertoli cells. As disruption of the cell adhesion, the lack of RNF20 in the Sertoli cells causes disorganization of the seminiferous tubules, and leads to the loss of the germ cells in the seminiferous tubules by the apoptosis pathway. The impairment of these molecular and cellular processes eventually causes spermatogenesis dysfunction and male infertility.

We uncover *Rnf20* as a new causative gene for spermatogenic failure and male infertility. The pathogenetic mechanism is involved in epigenetic regulation of the RNF20 in male infertility. RNF20 is an E3 ubiquitin ligase critical for mono-ubiquitination of histone H2B at lysine 120 [35, 36]. Knockout of *Rnf20* results in the loss of mono-ubiquitination of histone H2B at lysine 120 in the *Cldn11* gene, which disrupts its transcription elongation and expression in the Sertoli cells. H2BK120ub is an active epigenetic marker, which is known to regulate transcriptional elongation [43, 46, 47]. Thus, RNF20 regulates expression of the *Cldn11* gene through H2B modification of mono-ubiquitination and contributes to the cell-cell adhesion of the Sertoli cells and SSCs to establish the BTB in the seminiferous tubules, which ensures the Sertoli–germ cell crosstalk during spermatogenesis. Knockout of *Rnf20* disrupts the prime factor in the regulatory cascade RNF20-H2BK120ub-CLDN11 during spermatogenesis and eventually causes male infertility. It should be mentioned that knockout of *Rnf20* in the Sertoli cells also interferes with expression of the other genes, including *Dmrt1* and *Ctnna2*, in the GO terms spermatogenesis and cell adhesion in the Sertoli cells, probably via H2BK120ub. It is interesting to further explore the whole picture of regulation networks and functions of the RNF20-H2BK120ub in the Sertoli cells during spermatogenesis.

As a major component of the BTB, CLDN11 contributes to the cell-cell adhesion and crosstalk between the Sertoli cells and SSCs, which play important roles during spermatogenesis [29–31]. Phenotypes of male infertility in the *Amh-Rnf20*^*−/−*^ mice are basically similar to those of the *Cldn11* knockout mice. Deficiency of *Cldn11* resulted in the loss of the cell-cell adhesion and spermatogenesis was arrested before the spermatocyte stage [32]. Notably, the cell-cell adhesion in the seminiferous tubules of the *Amh-Rnf20*^*−/−*^ mice is seriously disrupted and key cell-adhesion molecules, α-Catenin, β-Catenin, and N-Cadherin, are disorganized in the seminiferous tubules in the *Amh-Rnf20*^*−/−*^ testis. It is well known that these cell-adhesion molecules are essential for the junction of intercellular adhesion [52–54]. Thus, the cascade RNF20-H2BK120ub-CLDN11 functions through the regulation of the cell-adhesion within the seminiferous tubules during spermatogenesis. Disruption of the cell adhesion causes disorganization of the seminiferous tubules, impairs normal processes of spermatogenesis, leads to the loss of both the spermatogonia and spermatocytes in the seminiferous tubules by the apoptotic cell death, and thus contributes to male infertility.

## Conclusions

This study shows that RNF20 is required for male fertility through regulation of H2B ubiquitination in the Sertoli cells. The Sertoli cells-specific knockout of *Rnf20* causes male infertility through regulation of the cascade RNF20-H2BK120ub-CLDN11 during spermatogenesis in the mouse testis.

## Methods

### Generation of the ***Amh-Rnf20***^***−/−***^ mice

Homozygous *Rnf20*^*Flox/Flox*^ mice were crossed with transgenic mice expressing the CRE recombinase under the control of the *Amh* promoter to generate the *Rnf20*^*Flox/Flox*^ and the *Amh-Rnf20*^*−/−*^ mice [45]. PCR primers were used for genotyping of the *Rnf20* and the *Amh* mice as follows: *Rnf20*: 5’ GCTGTAAGAGTTCTTAATGTATG 3’ and 5’ GGCTTGTCACACAAGCATGAGCATC 3’; *Amh*: 5’ TCCAATTTACTGACCGTACACCAA 3’ and 5’ CCTGTACCTGGCAATTTCGGCTA 3’. The PCR conditions were as follows: 94 °C for 3 min; 94 °C for 30 s, 60 °C for 30 s, and 72 °C for 30 s for 34 rounds; and 72 °C for 10 min.

All the animals were bred and raised in the Experimental Animal Center of Wuhan University and animal experiments and methods were performed in accordance with the relevant approved guidelines and regulations, as well as under the approval of the Ethics Committee of Wuhan University.

### Antibodies and reagents

The following primary antibodies were used: Anti-RNF20 (21625-1-AP, Proteintech Group, Rosemont, IL, USA), Anti-H2BK120ub (Cat# 5546s, Cell Signaling Technology, Danvers, MA, USA), Anti-β-ACTIN (Cat# 66009-1-Ig, Proteintech Group, Rosemont, IL, USA), Anti-SOX9 (Cat# 82,630 S, Cell Signaling Technology, Danvers, MA, USA), Anti-Caspase3 (Cat# 19677-1-AP, Proteintech Group, Rosemont, IL, USA), Anti-Claudin 11 (Cat# 36-4500, Thermo Fisher, Waltham, MA, USA), Anti-N-Cadherin (Cat# WL01047, Wanleibio, Shenyang, China), Anti-β-Catenin (Cat# 51067-2-AP, Proteintech Group, Rosemont, IL, USA), and Anti-α-Catenin (Cat# GTX111168, GeneTex, Texas, USA). DMRT1 antibody was provided by Professor Robin Lovell-Badge, MRC, UK.

The secondary antibodies were as follows: Horseradish peroxidase (HRP) conjugated-goat anti-Rabbit IgG (H + L) secondary antibody (Cat# 31,460) from Pierce Biotechnology Company, Rockford, IL, USA and horseradish peroxidase (HRP) conjugated-goat anti-Mouse IgG (H + L) secondary antibody (Cat# 31,430) from Invitrogen, Carlsbad, USA. The fluorescent TRITC-conjugated ImmunoPure goat anti-Rabbit IgG (H + L) (Cat# ZF-0316) was purchased from Feiyi Technology, Wuhan, Hubei, China. The fluorescent FITC-conjugated ImmunoPure goat anti-rabbit IgG (H + L) (Cat# ZF-0311) was from Feiyi Technology, Wuhan, Hubei, China. DAPI (Cat# C1002) was purchased from Beyotime Institute of Biotechnology, Nantong, Jiangsu, China.

### Histology analysis

Testes and epididymis were dissected immediately from mice after euthanasia. The tissues were fixed with 4% paraformaldehyde at 4 °C for overnight, then were embedded in paraffin and cut into a series of 5 μm sections using a paraffin slicer (CM1950, Leica, Germany). After deparaffinization and rehydration, the sections were stained with hematoxylin and eosin for histology analysis. Images were collected with automatic scanning microscope for tissue sections (DM6B, Leica, Germany).

### Western blot analysis

Western blot analysis was performed as previously described [55]. In brief, protein samples were extracted from the testes of adult mice, separated in 12% SDS-polyacrylamide gels, and transferred onto 0.45 μm PVDF membranes (IPVH00010, Millipore, Bedford, MA, USA). After blocked with 5% non-fat milk in TBST (20 mM Tris-HCl pH 7.5, 150 mM NaCl, 0.1% Tween 20) for 30 min at room temperature, the membranes were incubated with the primary antibodies (1:1000 dilution in 5% BSA) at 4 °C for overnight. The membranes were then washed with TBST 5 times for 10 min each time and incubated with HRP-labeled secondary antibody (1:5000 dilution in 5% non-fat milk) for 1 h at room temperature. After washed with TBST for 5 times, the signals were detected with the ECL kits (K-12,045-D50, Advansta, Menlo Park, USA).

### Immunofluorescence analysis

Testis tissues were fixed with 4% cold PFA for overnight and washed with PBS (pH 7.4) for 3 times. Then samples were embedded in paraffin and cut into a series of 5 μm sections using a paraffin slicer (CM1950, Leica, Germany). After deparaffinization and rehydration, antigen repairs for tissues sections were performed with 0.01 M citric acid solution (pH 6.0) under the condition of pressure (100 kPa) and temperature (105 °C) for 5 min, followed by natural cooling to room temperature. The tissues sections were treated with 1% Triton X-100 for 25 min and rinsed in PBS for 3 times. After blocking with 5% BSA for 20 min, the sections were incubated with primary antibody at 1:100 in 5% BSA at 4 °C for overnight. After washing in PBS, the sections were incubated with immunofluorescent secondary antibody at the dilution of 1:100 for 1 h at 37 °C, and washed in PBS for 3 times in the dark. The nuclei were stained with DAPI (Cat# C1002, Beyotime, Jiangsu) for 5 min and rinsed 3 times for 5 min in PBS. Images were collected immediately using confocal microscope (SP8, Leica, Germany).

### Mass spectrometry

Mass spectrometry analysis was performed as previously described [56]. To identify the levels of ubiquitination at H2BK120 in vivo, proteins were extracted from mouse testes with the genotype *Rnf20*^*Flox/Flox*^ or *Amh-Rnf20*^*−/−*^. Bradford method was used for protein quantification, and proteins of 20 µg for SDS-PAGE were stained with Coomassie brilliant blue R250. All proteins in each group were used for trypsin digestion with a ratio of protein to trypsin 50:1. After digestion for overnight at 37 °C and desalted with C18 SPE Cartridge, anti-K-ε-GG antibody beads (Cat# 5562 S, Cell Signal Technology, Bossdun, MA) were adopted to enrich ubiquitinated peptides. Then, the peptides were separated by HPLC liquid system and analyzed by Q-Exactive series mass spectrum (Q-Exactive HF, Thermo Fisher, Waltham, MA, USA). The original data of mass spectrometry were processed using MaxQuant software for subsequent identification and quantitative analysis.

### Isolation and culture of primary Sertoli cells of mouse testis

Briefly, testes from mice at 7 days after birth with the genotypes of the *Rnf20*^*Flox/Flox*^ and the *Amh-Rnf20*^*−/−*^ were removed and decapsulated. The seminiferous tubules were cut into small pieces and incubated in 0.2 mg/ml 200 µl IV collagenase (40510ES60, Yeasen Biotechnology, Shanghai) at 37 °C for 20 min with gently shaking intermittently. Then, the cells were collected by centrifugation at 2800 rpm for 5 min, washed once with PBS, resuspended in 0.25% trypsin (Cat#SH30042.01, HyClone, Beijing), incubated at 37 °C for 10 min with gentle shaking, and terminated trypsin digestion with medium containing serum. After filtration through a 300 meshes nylon, the cells were collected by centrifugation at 2800 rpm for 5 min. Then cells were resuspended in the medium (DMEM with 12% FBS), placed in 6-well plates, and cultured at 37 °C for overnight. After several passaging, the adherent cells were used for subsequent analysis.

### RNA-seq analysis

For RNA-seq analysis, total RNAs were extracted from the Sertoli cells of mouse testes at 7 days after birth (with the genotype *Rnf20*^*Flox/Flox*^ or *Amh-Rnf20*^*−/−*^), each group with three individuals, using the TRIzol Reagent (15596-026, Thermo Fisher, Waltham, MA, USA), according to manufacturer’s instructions. The RNA samples were treated with RNase-free DNase to remove DNA (M610A, Promega, Madison, WI, USA). After enriched using Oligo (dT), RNAs were broken up for cDNA library construct according to the manufacturer’s protocol. Then the cDNA libraries were sequenced using BGISEQ platform. To determine gene expression levels, clean reads from the Sertoli cells of the *Rnf20*^*Flox/Flox*^ or the *Amh-Rnf20*^*−/−*^ were mapped to the reference genome (GCF_000001635.26_GRCm38.p6) by HISAT and to the reference genes by Bowtie2. Fragments per kilo bases per million fragments (FPKM) values were calculated for each gene by RSEM. Differentially expressed genes were defined with |Log_2_FC|>1 and FDR < 0.001.

### Quantitative real-time PCR

Total RNA samples were isolated from testes of adult mice using TRIzol reagent (Invitrogen, CarIsbad, CA, USA) according to the manufacturer’s instructions, and then were treated with RNase-free DNase (M610A, Promega, Madison, WI, USA). Using the MMLV system (M1701, Promega), cDNAs were reversely transcribed from the RNAs. SYBR Green qPCR Mix (Q712, Vazyme, Nanjing, China) was used for quantitative real-time PCR amplification in a StepOne real-time PCR system (Applied Biosystems, USA). The primers of *Cldn11* and *Hprt* were listed in Additional file 1: Table S1.

### ChIP-PCR analysis

The testis samples were dissected immediately from adult mice after euthanasia and cut into small pieces. The testis samples were then cross-linked by 1% formaldehyde and terminated by glycine with a final concentration of 0.125 M. After homogenized, the samples were resuspended in lysis buffer (50 mM Tris-HCl pH 8.0, 5 mM EDTA, 0.1% SDS) and the genomic DNAs were sonicated into fragments of 200–500 bp (Bioruptor Pico sonicator, high, 25cycle, 30s on/off). The DNA samples of 25 µg were incubated with protein A/G beads (36403ES25, Yeasen Biotechnology, Shanghai) conjugated with 2 µg primary antibody or IgG at 4 °C for overnight. After washed, the beads were used to extract genomic DNA with phenol-chloroform extraction methods. The primer sequences were described in Additional file 1: Table S2.

### TUNEL assays

The testis samples were embedded in paraffin and cut into a series of 5 μm sections using a paraffin slicer (CM1950, Leica, Germany). Antigen repairs for tissues sections were performed as described in the section immunofluorescence analysis. For TUNEL (terminal deoxynucleotidyl transferase-mediated dUTP Nick-End Labeling) assays, one step TUNEL apoptosis assay kit (C1086, Beyotime) was used according to the manufacturer’s protocol. The testis tissues sections were permeabilized with 20 µg/ml proteinase K (P2308, Sigma-Aldrich) for 20 min at room temperature, and then the sections were incubated with the FITC-labeling reaction mix containing TdT and fluorescein-dUTP for 1 h at 37 °C. The nuclei were stained by DAPI for 5 min. Images were captured by a confocal fluorescent microscope (FV1000, Olympus, Tokyo, Japan).

### Statistical analysis

The data are presented as the means ± standard at least three independent experiments. Statistical comparisons were made using Student’s t-test when comparing two groups. One-way ANOVA was performed for comparisons with more than two groups. Statistics analysis was performed using GraphPad Prism 7 software package. For significant test, p < 0.05 was considered to be statistically significant [57].

## Electronic supplementary material

Below is the link to the electronic supplementary material.

Supplementary Material 1

## Data Availability

The accession number for the sequence reported in this paper is GEO GSE225232.

## Declarations

### Supporting information

Additional files include 3 figures and 2 tables.

## References

[1] Ombelet W, Cooke I, Dyer S, Serour G, Devroey P (2008). Infertility and the provision of infertility medical services in developing countries. Hum Reprod Update.

[2] Inhorn MC, Patrizio P (2015). Infertility around the globe: new thinking on gender, reproductive technologies and global movements in the 21st century. Hum Reprod Update.

[3] Farquhar CM, Bhattacharya S, Repping S, Mastenbroek S, Kamath MS, Marjoribanks J, Boivin J (2019). Female subfertility. Nat Rev Dis Primers.

[4] Cheng H, Shang D, Zhou R (2022). Germline stem cells in human. Signal Transduct Target Ther.

[5] Pan MM, Hockenberry MS, Kirby EW, Lipshultz LI (2018). Male infertility diagnosis and treatment in the era of in Vitro fertilization and intracytoplasmic sperm injection. Med Clin North Am.

[6] Wang RX, Fu C, Yang YP, Han RR, Dong Y, Dai RL, Liu RZ (2010). Male infertility in China: laboratory finding for AZF microdeletions and chromosomal abnormalities in infertile men from northeastern China. J Assist Reprod Genet.

[7] Adamczewska D, Slowikowska-Hilczer J, Marchlewska K, Walczak-Jedrzejowska R (2020). Features of gonadal dysgenesis and Leydig cell impairment in testes with sertoli cell-only syndrome. Folia Histochem Cytobiol.

[8] Sharma A, Jain M, Halder A, Kaushal S (2021). Identification of genomic imbalances (CNVs as well as LOH) in sertoli cell only syndrome cases through cytoscan microarray. Gene.

[9] Lin YH, Chuang L, Lin YM, Lin YH, Teng YN, Kuo PL (2005). Isochromosome of Yp in a man with sertoli-cell-only syndrome. Fertil Steril.

[10] Stouffs K, Gheldof A, Tournaye H, Vandermaelen D, Bonduelle M, Lissens W, Seneca S. Sertoli Cell-Only Syndrome: Behind the Genetic Scenes. Biomed Res Int. 2016; 2016. 10.1155/2016/6191307 PMC4746273 26925412

[11] Shahid M, Dhillon VS, Khalil HS, Sexana A, Husain SA (2011). Associations of Y-chromosome subdeletion gr/gr with the prevalence of Y-chromosome haplogroups in infertile patients. Eur J Hum Genet.

[12] Foresta C, Ferlin A, Garolla A, Moro E, Pistorello M, Barbaux S, Rossato M (1998). High frequency of well-defined Y-chromosome deletions in idiopathic sertoli cell-only syndrome. Hum Reprod.

[13] Kamp C, Huellen K, Fernandes S, Sousa M, Schlegel PN, Mielnik A, Kleiman S, Yavetz H, Krause W, Küpker W (2001). High deletion frequency of the complete AZFa sequence in men with sertoli-cell-only syndrome. Mol Hum Reprod.

[14] Vogt PH, Edelmann A, Kirsch S, Henegariu O, Hirschmann P, Kiesewetter F, Köhn FM, Schill WB, Farah S, Ramos C (1996). Human Y chromosome azoospermia factors (AZF) mapped to different subregions in Yq11. Hum Mol Genet.

[15] Koc G, Ozdemir AA, Girgin G, Akbal C, Kirac D, Avcilar T, Guney AI (2019). Male infertility in sertoli cell-only syndrome: an investigation of autosomal gene defects. Int J Urol.

[16] Miyamoto T, Bando Y, Koh E, Tsujimura A, Miyagawa Y, Iijima M, Namiki M, Shiina M, Ogata K, Matsumoto N (2016). A PLK4 mutation causing azoospermia in a man with sertoli cell-only syndrome. Andrology.

[17] Zhang Y, Li P, Liu N, Jing T, Ji Z, Yang C, Zhao L, Tian R, Chen H, Huang Y (2021). Novel bi-allelic variants of FANCM cause sertoli cell-only syndrome and non-obstructive azoospermia. Front Genet.

[18] Arafat M, Zeadna A, Levitas E, Har Vardi I, Samueli B, Shaco-Levy R, Dabsan S, Lunenfeld E, Huleihel M, Parvari R (2020). Novel mutation in USP26 associated with azoospermia in a sertoli cell-only syndrome patient. Mol Genet Genomic Med.

[19] Miyamoto T, Iijima M, Shin T, Minase G, Ueda H, Okada H, Sengoku K (2018). CUL4B mutations are uncommon in japanese patients with sertoli-cell-only syndrome and azoospermia. J Obstet Gynaecol.

[20] Lardone MC, Argandona F, Lorca M, Piottante A, Florez M, Palma C, Ebensperger M, Castro A (2018). Leydig cell dysfunction is associated with post-transcriptional deregulation of CYP17A1 in men with sertoli cell-only syndrome. Mol Hum Reprod.

[21] Almeida C, Correia S, Rocha E, Alves A, Ferraz L, Silva J, Sousa M, Barros A (2013). Caspase signalling pathways in human spermatogenesis. J Assist Reprod Genet.

[22] Lee SJ, Park J, Lee DJ, Otsu K, Kim P, Mizuno S, Lee MJ, Kim HY, Harada H, Takahashi S (2021). Mast4 knockout shows the regulation of spermatogonial stem cell self-renewal via the FGF2/ERM pathway. Cell Death Differ.

[23] Wang Z, Xu X, Li JL, Palmer C, Maric D, Dean J (2019). Sertoli cell-only phenotype and scRNA-seq define PRAMEF12 as a factor essential for spermatogenesis in mice. Nat Commun.

[24] Ghanami Gashti N, Sadighi Gilani MA, Abbasi M (2021). Sertoli cell-only syndrome: etiology and clinical management. J Assist Reprod Genet.

[25] Chen C, Ouyang W, Grigura V, Zhou Q, Carnes K, Lim H, Zhao GQ, Arber S, Kurpios N, Murphy TL (2005). ERM is required for transcriptional control of the spermatogonial stem cell niche. Nature.

[26] Oatley JM, Brinster RL (2012). The germline stem cell niche unit in mammalian testes. Physiol Rev.

[27] Washburn RL, Hibler T, Kaur G, Dufour JM (2022). Sertoli cell Immune Regulation: a double-edged Sword. Front Immunol.

[28] Mital P, Kaur G, Dufour JM (2010). Immunoprotective sertoli cells: making allogeneic and xenogeneic transplantation feasible. Reproduction.

[29] McCabe MJ, Foo CF, Dinger ME, Smooker PM, Stanton PG (2016). Claudin-11 and occludin are major contributors to sertoli cell tight junction function, in vitro. Asian J Androl.

[30] Smith BE, Braun RE (2012). Germ cell migration across sertoli cell tight junctions. Science.

[31] Gow A, Southwood CM, Li JS, Pariali M, Riordan GP, Brodie SE, Danias J, Bronstein JM, Kachar B, Lazzarini RA (1999). CNS myelin and sertoli cell tight junction strands are absent in Osp/claudin-11 null mice. Cell.

[32] Mazaud-Guittot S, Meugnier E, Pesenti S, Wu X, Vidal H, Gow A, Le Magueresse-Battistoni B (2010). Claudin 11 deficiency in mice results in loss of the sertoli cell epithelial phenotype in the testis. Biol Reprod.

[33] Chiba K, Yamaguchi K, Ando M, Miyake H, Fujisawa M (2012). Expression pattern of testicular claudin-11 in infertile men. Urology.

[34] Kanatsu-Shinohara M, Ogonuki N, Matoba S, Ogura A, Shinohara T (2020). Autologous transplantation of spermatogonial stem cells restores fertility in congenitally infertile mice. Proc Natl Acad Sci U S A.

[35] Hwang WW, Venkatasubrahmanyam S, Ianculescu AG, Tong A, Boone C, Madhani HD (2003). A conserved RING finger protein required for histone H2B monoubiquitination and cell size control. Mol Cell.

[36] Wood A, Krogan NJ, Dover J, Schneider J, Heidt J, Boateng MA, Dean K, Golshani A, Zhang Y, Greenblatt JF (2003). Bre1, an E3 ubiquitin ligase required for recruitment and substrate selection of Rad6 at a promoter. Mol Cell.

[37] Fuchs G, Oren M (2014). Writing and reading H2B monoubiquitylation. Biochim Biophys Acta.

[38] So CC, Ramachandran S, Martin A (2019). E3 ubiquitin ligases RNF20 and RNF40 are required for double-stranded break (DSB) repair: evidence for monoubiquitination of histone H2B lysine 120 as a Novel Axis of DSB signaling and repair. Mol Cell Biol.

[39] Shema E, Tirosh I, Aylon Y, Huang J, Ye C, Moskovits N, Raver-Shapira N, Minsky N, Pirngruber J, Tarcic G (2008). The histone H2B-specific ubiquitin ligase RNF20/hBRE1 acts as a putative tumor suppressor through selective regulation of gene expression. Genes Dev.

[40] Liang Q, Xia W, Li W, Jiao J (2018). RNF20 controls astrocytic differentiation through epigenetic regulation of STAT3 in the developing brain. Cell Death Differ.

[41] Fuchs G, Shema E, Vesterman R, Kotler E, Wolchinsky Z, Wilder S, Golomb L, Pribluda A, Zhang F, Haj-Yahya M (2012). RNF20 and USP44 regulate stem cell differentiation by modulating H2B monoubiquitylation. Mol Cell.

[42] Nakamura K, Kato A, Kobayashi J, Yanagihara H, Sakamoto S, Oliveira DV, Shimada M, Tauchi H, Suzuki H, Tashiro S (2011). Regulation of homologous recombination by RNF20-dependent H2B ubiquitination. Mol Cell.

[43] Wu C, Cui Y, Liu X, Zhang F, Lu LY, Yu X (2020). The RNF20/40 complex regulates p53-dependent gene transcription and mRNA splicing. J Mol Cell Biol.

[44] Liang X, Tao C, Pan J, Zhang L, Liu L, Zhao Y, Fan Y, Cao C, Liu J, Zhang J (2021). Rnf20 deficiency in adipocyte impairs adipose tissue development and thermogenesis. Protein Cell.

[45] Xu Z, Song Z, Li G, Tu H, Liu W, Liu Y, Wang P, Wang Y, Cui X, Liu C (2016). H2B ubiquitination regulates meiotic recombination by promoting chromatin relaxation. Nucleic Acids Res.

[46] Fuchs G, Hollander D, Voichek Y, Ast G, Oren M (2014). Cotranscriptional histone H2B monoubiquitylation is tightly coupled with RNA polymerase II elongation rate. Genome Res.

[47] Pavri R, Zhu B, Li G, Trojer P, Mandal S, Shilatifard A, Reinberg D (2006). Histone H2B monoubiquitination functions cooperatively with FACT to regulate elongation by RNA polymerase II. Cell.

[48] Liu P, Jenkins NA, Copeland NG (2003). A highly efficient recombineering-based method for generating conditional knockout mutations. Genome Res.

[49] Rahmoun M, Lavery R, Laurent-Chaballier S, Bellora N, Philip GK, Rossitto M, Symon A, Pailhoux E, Cammas F, Chung J (2017). In mammalian foetal testes, SOX9 regulates expression of its target genes by binding to genomic regions with conserved signatures. Nucleic Acids Res.

[50] Matson CK, Murphy MW, Griswold MD, Yoshida S, Bardwell VJ, Zarkower D (2010). The mammalian doublesex homolog DMRT1 is a transcriptional gatekeeper that controls the mitosis versus meiosis decision in male germ cells. Dev Cell.

[51] Krausz C, Riera-Escamilla A (2018). Genetics of male infertility. Nat Rev Urol.

[52] Newton SC, Blaschuk OW, Millette CF (1993). N-cadherin mediates sertoli cell-spermatogenic cell adhesion. Dev Dyn.

[53] van der Wal T, van Amerongen R (2020). Walking the tight wire between cell adhesion and WNT signalling: a balancing act for beta-catenin. Open Biol.

[54] Kobielak A, Fuchs E (2004). Alpha-catenin: at the junction of intercellular adhesion and actin dynamics. Nat Rev Mol Cell Biol.

[55] Sheng Y, Song Y, Li Z, Wang Y, Lin H, Cheng H, Zhou R (2018). RAB37 interacts directly with ATG5 and promotes autophagosome formation via regulating ATG5-12-16 complex assembly. Cell Death Differ.

[56] Lai F, Cheng Y, Zou J, Wang H, Zhu W, Wang X, Cheng H, Zhou R (2021). Identification of histone modifications reveals a role of H2b monoubiquitination in Transcriptional Regulation of dmrt1 in Monopterus albus. Int J Biol Sci.

[57] Hong Q, Li C, Ying R, Lin H, Li J, Zhao Y, Cheng H, Zhou R (2019). Loss-of-function of sox3 causes follicle development retardation and reduces fecundity in zebrafish. Protein Cell.

