# Supplementary material for: RNF20 is required for male fertility through regulation of H2B ubiquitination in the Sertoli cells
Source: Cell Biosci. 2023 Apr 6;13:71. doi: 10.1186/s13578-023-01018-2 (PMC10080854; doi:10.1186/s13578-023-01018-2)
Supplement: Supplementary file 1 — Supplementary Material 1 [file 13578_2023_1018_MOESM1_ESM.pdf]

Additional file 1

fig. S1. Oogenesis is not impaired in *Amh-Rnf20*$^{-/-}$ mice. **a** H&E staining of ovaries from adult mice, *Rnf20*$^{Flox/Flox}$, *Amh-Rnf20*$^{+/-}$, and *Amh-Rnf20*$^{-/-}$. Scale bar, 50 µm. **b** Percentage of primary, secondary, mature, and atretic follicles after H&E staining on ovary sections in *Rnf20*$^{Flox/Flox}$, *Amh-Rnf20*$^{+/-}$, and *Amh-Rnf20*$^{-/-}$. Left panel, female mice at one-month after birth; Right panel, female mice at two-months after birth (n=3).

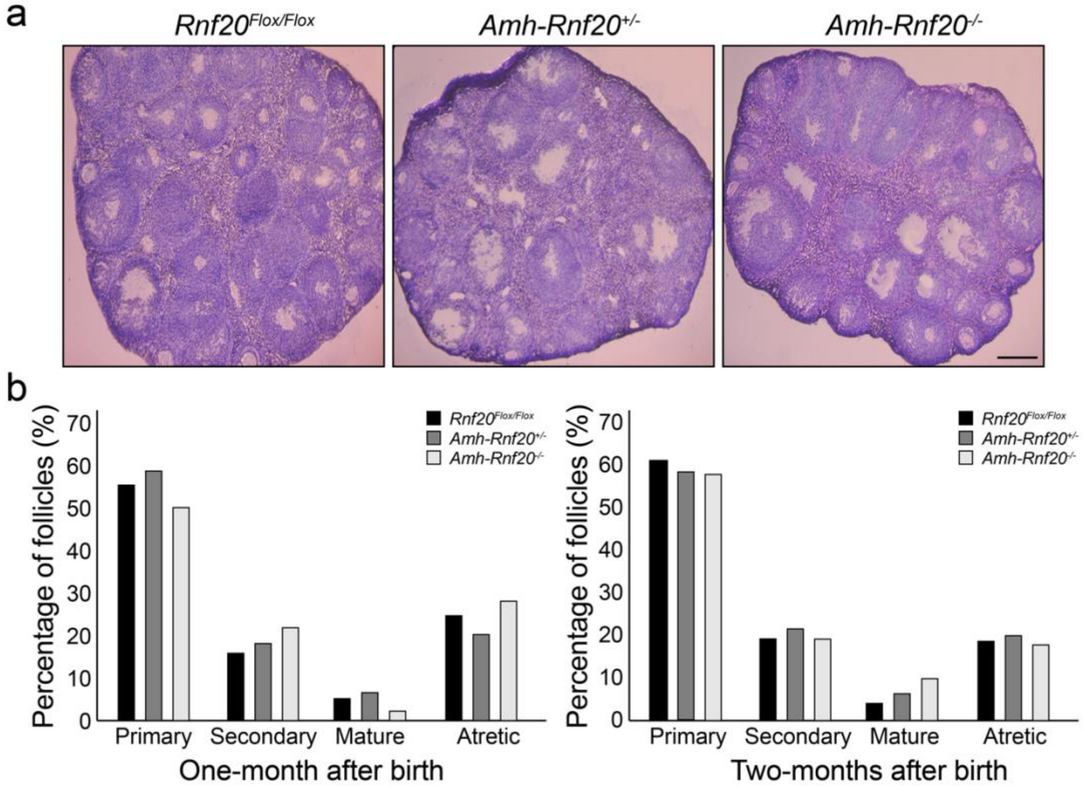

fig. S2. H2BK120ub is deficient in the Sertoli cells in *Amh-Rnf20*$^{-/-}$ mice at different ages during spermatogenesis. **a, b** Immunofluorescent analysis of the localization of SOX9, H2BK120ub, and DMRT1 in seminiferous tubules on serial paraffin-sections of testes in *Rnf20*$^{Flox/Flox}$ and *Amh-Rnf20*$^{-/-}$ at 7, 14, 16, 18, 21, and 28 days after birth and in adult mice. The nuclei were stained with DAPI and TRITC signals represent the location of H2BK120ub, while FITC signals represent the location of SOX9 or DMRT1, respectively. The white squares in the merge panels correspond to the enlarged regions. Sn, Sertoli cells. Scale bar, 25 µm.

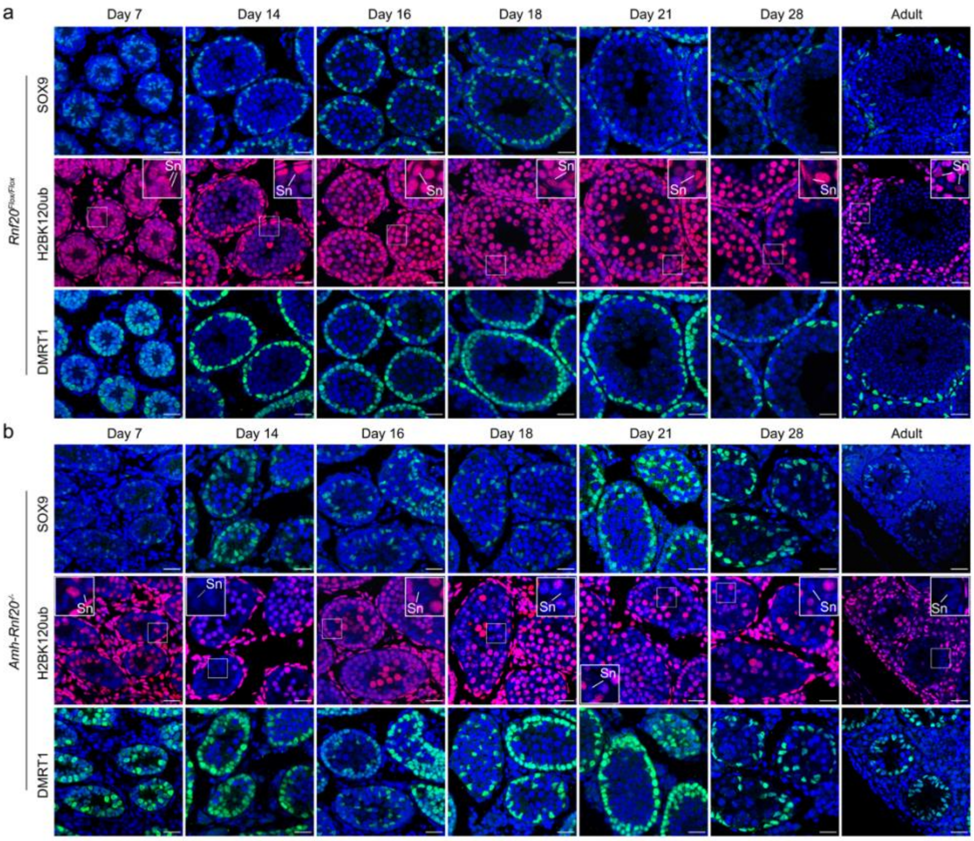

fig. S3 ChIP-PCR analysis of the enrichment levels of H2BK120ub in different regions of *Cldn11*. **a** Schematic diagram of relative positions of primers in *Cldn11* in the ChIP assays. **b** ChIP analysis showed that H2BK120ub could bind to the exon regions but seldomly in the promoter of *Cldn11 in vivo*.

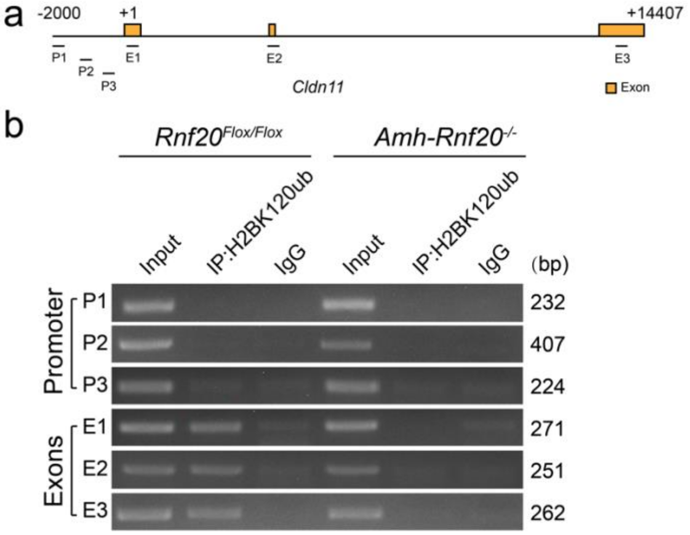

Table S1. Primer sequences for quantitative real-time PCR

Name	Primer sequence
Cldn11-Forward	5’ GACATCCTCATCCTTCCAGGCTAC 3’
Cldn11-Reverse	5’ CCCACCAGGCACATCACAG 3’
Rnf20-Forward	5’ GTTCGCAAGGAGTATGAG 3’
Rnf20-Reverse	5’ CATCCCGTTTGGACTTA 3’
Hprt-Forward	5’ TTGCTGACCTGCTGGATTA 3’
Hprt-Reverse	5’ TAGGCTTTGTATTTGGCTTT 3’

Table S2. Primer sequences for ChIP-PCR analysis

Name	Primer sequence
Cldn11-P1-Forward	5’ TGTCCTCTGACCTCTATGCG 3’
Cldn11- P1-Reverse	5’ GGTGCTGTTGATTGACAAGTTAG 3’
Cldn11-P2-Forward	5’ TCCCGTGTCCTGCTCTATC 3’
Cldn11- P2-Reverse	5’ TCCCTCCTAAACTTCTACCAA 3’
Cldn11-P3-Forward	5’ GTTGGTAGAAGTTTAGGAGGGA 3’
Cldn11- P3-Reverse	5’ TGCGGGTGTAGAAGGGA 3’
Cldn11-E1-Forward	5’ GATTGGCATCATCGTCACA 3’
Cldn11-E1-Reverse	5’ AGGTCTCTGAGTCTCCAAAC 3’
Cldn11-E2-Forward	5’ ATGATTGCTGCCTCCGTT 3’
Cldn11-E2-Reverse	5’ CTGTCGCCAGTGGGTTC 3’
Cldn11-E3-Forward	5’ CTCGCTGTACGCAGGTTG 3’
Cldn11-E3-Reverse	5’ CGGGGCAGGATACTTTCTA 3’
